# The Thaayorre think of Time Like They Talk of Space

**DOI:** 10.3389/fpsyg.2012.00300

**Published:** 2012-08-28

**Authors:** Alice Gaby

**Affiliations:** ^1^Linguistics, School of Languages, Cultures and Linguistics, Monash UniversityClayton, VIC, Australia; ^2^Department of Linguistics, University of CaliforniaBerkley, CA, USA

**Keywords:** time, space, metaphor, metonymy, frames of reference, Pama-Nyungan, Australian Aboriginal

## Abstract

Around the world, it is common to both talk and think about time in terms of space. But does our conceptualization of time simply reflect the space/time metaphors of the language we speak? Evidence from the Australian language Kuuk Thaayorre suggests not. Kuuk Thaayorre speakers do not employ active spatial metaphors in describing time. But this is not to say that spatial language is irrelevant to temporal construals: non-linguistic representations of time are shown here to covary with the linguistic system of describing space. This article contrasts two populations of ethnic Thaayorre from Pormpuraaw – one comprising Kuuk Thaayorre/English bilinguals and the other English-monolinguals – in order to distinguish the effects of language from environmental and other factors. Despite their common physical, social, and cultural context, the two groups differ in their representations of time in ways that are congruent with the language of space in Kuuk Thaayorre and English, respectively. Kuuk Thaayorre/English bilinguals represent time along an absolute east-to-west axis, in alignment with the high frequency of absolute frame of reference terms in Kuuk Thaayorre spatial description. The English-monolinguals, in contrast, represent time from left-to-right, aligning with the dominant relative frame of reference in English spatial description. This occurs in the absence of any east-to-west metaphors in Kuuk Thaayorre, or left-to-right metaphors in English. Thus the way these two groups think about time appears to reflect the language of space and not the language of time.

## Introduction

Time and space are intimately related in language, thought, and experience. When viewing a moving object, for instance, we link the set of spatial relationships between that object and its background to a set of moments in time. But though we can see the changing location of the object, we lack any direct sensory access to time. And so it is natural that metaphorical descriptions take time as their target and describe it in terms of space. Indeed, such metaphors are so widespread they have been claimed to be universal (e.g., Traugott, 1978; Lakoff and Johnson, 1980; Alverson, 1994; Haspelmath, 1997; Evans, 2004). The connection between space and time runs deeper than language: numerous studies have shown that speakers construct mental representations of time in terms of space, with these construals being sensitive to linguistic manipulation (Boroditsky, 2000, 2001; Boroditsky and Ramscar, 2002; Walsh, 2003; Casasanto and Boroditsky, 2008; Casasanto et al., 2010).

Yet most of the research on space/time mappings in language and thought has focused on languages that primarily encode spatial relationships according to a “relative” frame of reference (calculated from the perspective of some external viewer. See, for example, the range of studies of space – time mapping in English, Chinese, German, and Hebrew, including Yu, 1998; Boroditsky, 2000; Gentner, 2001; Torralbo et al., 2006; Santiago et al., 2007; Tenbrink, 2007; Casasanto and Boroditsky, 2008; Weger and Pratt, 2008; Bender et al., 2010). This relative frame is just one of the three basic systems for describing spatial relations (cf., e.g., Levinson, 2003; Levinson and Wilkins, 2006). There is a large body of research demonstrating cognitive consequences of speaking a language with a dominant relative frame of reference (anchored to a viewer’s perspective, as with the terms *left* and *right*) as opposed to an “absolute” one (anchored to a set of coordinates independent of any observer, as with the terms *north* and *east*, see, e.g., Levinson, 1997, 2001; Munnich and Landau, 2003; Majid et al., 2004; Haun et al., 2011). Indeed, the relationship between language and spatial cognition has proven fertile – and hotly contested – ground in the debate over whether language shapes thought (e.g., Levinson et al., 2002; Li and Gleitman, 2002; Li et al., 2011; Pollian and Bohnemeyer, 2011). Given the widespread mapping of space to time in conceptual metaphor, then, we might expect to find an analogous split between relative and absolute temporal representations.

Recently, Boroditsky and Gaby (2010) investigated how time is represented by speakers of Australian languages with an absolute spatial reference system. They found these speakers to represent time along the absolute east-west axis, a radical departure from the egocentric relative conceptualizations of time previously documented. But is this absolute representation of time necessarily a product of language? The frequent use of absolute spatial language might feed representations of time in absolute terms, but so too may other aspects of cultural and physical environment (as explored further under Discussion). The influence of the physical environment is accorded particular prominence in Li and Gleitman’s (2002) critique of studies showing spatial language and spatial cognition to covary. The influence of writing direction on how time is represented, meanwhile, is well-established. Tversky et al. (1991), for example, show people who write from left-to-right to map the past onto the left and the future onto the right. Bergen and Chan Lau (2012) further find temporal representations to mirror different writing directions (top-to-bottom vs. left-to-right) even where language and culture are held constant.

This article turns to consider the effects of language on temporal representations, while holding environmental/social context and writing direction constant. By contrasting two speech populations within a single community, the present study is well placed to examine the effects of language without confounding social and environmental variables. Through two experimental tasks (Eliciting Improvised Representations of Time and Results) – with corroboration from the informal observation of gesture (Traditional Non-linguistic Representations of Time) – it shows ethnic Thaayorre to represent time differently depending on whether they speak only English or are bilingual in English and Kuuk Thaayorre. But while the east-to-west representations of time made by Kuuk Thaayorre speakers reflect their absolute description of spatial relationships (described under The Language of Space), they do not reflect how they speak about time (described under The Language of Time). Absolute space/time metaphors are entirely absent from Kuuk Thaayorre, just as spoken English lacks any metaphors to parallel the left-to-right temporal representations of time constructed by its speakers. Given the finding that spatial language and temporal conceptualization covary, the Discussion section below considers the evidence for – and problems with – a causal relationship between language and thought.

## Materials and Methods

### The language and its speakers

Kuuk Thaayorre is a Paman language spoken by more than 200 people in the aboriginal community of Pormpuraaw, on the west coast of Cape York Peninsula, Australia. Only a handful of children are currently acquiring Kuuk Thaayorre as a first language, and all but a couple of Kuuk Thaayorre speakers are also fluent in English. Although Pormpuraaw is located on Thaayorre land, speakers of other indigenous languages moved to the area when an Anglican mission was established there in 1938.

Though most Kuuk Thaayorre speakers prefer to use this language in the home and for social interaction, English is the language of most official institutions in the community (such as the school, church, store, police station, cultural center, and council), which are generally run by outsiders.

There is no traditional system for writing Kuuk Thaayorre. A number of Kuuk Thaayorre orthographies (all written from left-to-right) have been in existence since the 1960s. All of the participants in the experiments reported under Results were literate in English (cf., Language, its Conspirators and Competitors), and most also had limited literacy in Kuuk Thaayorre.

### The language of space

The canon of work on spatial reference has identified radically different systems (or “frames of reference”), classifying languages according to the predominant system (cf., e.g., Wassmann and Dasen, 1998; Levinson, 2003; Levinson and Wilkins, 2006; O’Meara and Pérez Báez, 2011). English speakers predominantly employ the “relative” and “intrinsic” frames of reference. The reference of terms used within a relative frame (such as *left* and *right* in the example to follow) must be calculated according to a viewer’s perspective; if I am told that *the glass on the left is filled with wine, the glass on the right with poison*, I would want to know my instructor’s vantage point before choosing my drink. Intrinsic terms (such as *behind* and *in front of* as used in the following example) are insensitive to the viewer’s perspective, instead being calculated according to inherent features of the reference object. So if I am told that *the glass of poison is in the middle of the table and the glass of wine is at the edge*, I can make my choice without paying attention to anyone’s vantage point, since the middle and edges of the table are defined by the internal properties of the table itself. The third, “absolute” frame of reference has more restricted use in English. Absolute terms (such as *north* and *east* below) are anchored to a fixed set of coordinates independent of any observer’s viewpoint and insensitive to the features of any reference object. English speakers rarely if ever use such terms to describe non-geographical, small-scale arrays (e.g., *the glass of poison is to the east of the glass of wine*), though there are plenty of languages around the world whose speakers do (cf., Pederson et al., 1998; Levinson, 2003; Levinson and Wilkins, 2006). Kuuk Thaayorre speakers are among them, having at their disposal dozens of absolute terms, a handful of intrinsic terms, and no terms invoking a relative frame of reference. Among the intrinsic set are two terms referring to the left and right hands of a person or animal and extending to the immediately adjacent areas. Though their English glosses suggest that these terms might in fact be relative, they are always anchored by the inherent left and right hemispheres of the body in question (rather than the left and right sides projected by an external viewer). So if we translated the sentence *Jan is standing to the right of the Statue of Liberty* into Kuuk Thaayorre, it would only be true if Jan was to the statue’s southwest (since the statue faces roughly southeast). Furthermore, the statement remains true regardless of where I am standing, even though Jan is in my left visual field if I am viewing them from the southeast. A fuller list of intrinsic spatial terms is given in Gaby (2006).

Kuuk Thaayorre absolute terms are employed with extremely high frequency in describing everything from small-scale arrangements of objects to geographical locations. These terms comprise the core of the directional adverb paradigm. The six absolute directional root forms refer to the four cardinal directions and the north and south banks of the nearby Edward River. The terms -*kaw* “east” and -*kuw* “west” are defined by the sun’s trajectory, while the terms roughly translated as “∼north” and “∼south” (-*ungkarr* and -*iparr*, respectively), more accurately align with an axis defined by the local coastline, forming an axis rotated almost 45° clockwise from that perpendicular to east-west. The directional roots are obligatory preceded by two prefixes, the first marking distance from the deictic center, and the second optionally encoding motion and/or orientation. The root may also be followed by up to two suffixes. The first further specifies the direction, for example by adding the suffix -*uw* to the stem *ii-ø-parr* “in the ∼south” to create *ii-ø-parr-uw* “in the ∼southwest.” The second adds the river as a relevant reference point, usually the start- or endpoint of motion, as seen in example (1).

**Table:** 

(1)	*ngay*	*ii-rr-iparr-op*	*yancm*
	1sg (nom)	there-toward-south-river	go:p.ipfv
	‘I went down∼south, riverward’ (AJ27Jan04 Conversation)		

### The language of time

The Kuuk Thaayorre temporal lexicon includes numerous labels for portions of the diurnal and seasonal cycles, as well as deictic terms for “yesterday,” “tomorrow,” “next time,” “soon,” “long ago,” and so on. The deictic temporal terms might be argued to employ a relative frame of reference, since they are anchored to an experiencer’s perspective. It is important to note, however, that they are not inherently yoked to any particular spatial frame of reference; “soon” and “long ago,” for example, could in theory occupy any number of positions with respect to each other and/or the deictic center. Though absolute space-time metaphors are absent, two times of the day are labeled with reference to the sun. The late morning to noon period is labeled *raak pung putpun* “the time when the sun is at the top” (literally, “time/place sun on. top”), while at least one speaker referred to the sunset period as *pung kaalkurrc* “(the time when the) sun (is) cold.” Though such terms are not metaphorical *per se*, they do anchor temporal reference to the absolute frame by using the position of the sun as an index for time. While the terms for times of day are apparently conventional, they are extremely infrequently used. Indeed, I have only encountered such expressions in elicitation contexts in response to direct solicitation. They are entirely absent from the texts and conversations I have recorded, in stark contrast with the frequent use of the deictic temporal terms mentioned above.

Space-time mappings are extremely few in number but high in frequency. Most obvious here is the use of a single term, *raak*, to refer to both “place” and “time” (as well as the “ground,” “dirt,” “earth,” and more). Likewise, *kanpa* encodes both the intrinsic relation “in front of” and temporal priority. No relative space-time mappings are attested, nor are the intrinsic terms *punth thak* “left-hand side” and *punth mal* “right-hand side” ever used with temporal meaning. No active space-time metaphors are apparent, beyond the lexical ambiguities already noted. It is not *a priori* clear whether these ambiguous terms spring from an original domain mapping or conventional metaphor (cf., Croft, 1993; Gentner, 2001). The results discussed below, however, are suggestive of a domain mapping from space to time (cf. Discussion).

### Traditional non-linguistic representations of time

The Kuuk Thaayorre traditionally kept track of time’s passage by monitoring the cycles of the moon and by the various seasonal changes in flora, fauna, and weather. One of my consultants mentions tying knots in a piece of string in order to count months. He states that these knots were not “read” from left-to-right or any other particular orientation, they were simply counted. Other systems of marking time periods on the body were widespread in Aboriginal Australia and may well have been employed in Pormpuraaw. For instance, Harris (1982, p. 165) writes of Ngalkbon message bearers having “their actual bodies marked to indicate, for example, that a particular event was planned for a specific day in the lunar cycle,” with 28 successive positions on the body corresponding to the phases of the moon.

A detailed ethnography of pointing and other gestures remains to be conducted. Even in its absence, however, it is clear that Kuuk Thaayorre speakers often point to the (imagined) position of the sun in order to indicate times of day (e.g., directly upward when referring to noon, westward when referring to the evening). I have also observed people pointing eastward to refer to the more distant past (e.g., 40 years earlier). Though these data are only suggestive, there is other evidence that the spatial representations constructed in experimental contexts have structural analogs in gesture. Kita et al. (2001), for instance, find the different systems of spatial gesture among two Mayan populations to mirror differences in how the two groups perform in a pattern-matching task. Furthermore, Kita, Danziger, and Stolz note that while the Yucatec Mayans represent the passage of time with right-to-left lateral gestures, such gestures are entirely absent among Mopan speakers (the single temporal gesture recorded from a Mopan speaker involved near-to-far movement along the sagittal axis). Also consider Le Guen’s (2011) contention regarding Yucatec Maya that a preferred frame of reference only emerges through the concurrent study of language and gesture, and is not evident in language alone (cf. also Le Guen and Pool Balam, 2012). In other speech communities that employ an absolute spatial reference system, Levinson (2002) notes that systematic gestures “sometimes (locate) the past in, for example, a southerly direction and the future in the north.” The Aymara also demonstrate an alignment of temporal gestures with spoken metaphors of time in terms of space (Núñez and Sweetser, 2006).

Sand drawings were and remain a common visual accompaniment to Thaayorre oral narratives (as is common around Australia). These represent participants, locations, and trajectories from a bird’s eye perspective, internally consistent within the absolute frame of reference. Any representation of time in sand drawings is iconic, with earlier events being drawn before later events, fast motion being drawn more speedily than slow ones. Sequentially related events occurring in the same location are depicted by erasing the prior event and drawing the later event in its place. As David Nash (email: January 5, 2011) points out, erasure may also be used to mark major episodic breaks, which frequently have temporal significance.

### Eliciting improvised representations of time

To probe how Kuuk Thaayorre speakers conceptualize time, I – in collaboration with Lera Boroditsky – ran two experiments designed to elicit spatial representations of time. In the first of these experimental tasks, participants were asked to arrange sets of cards depicting a temporal sequence in order from earliest to latest. For example, a card with a photo of a crocodile egg might be followed by a photo of a crocodile hatching, followed by a juvenile crocodile, followed by a mature crocodile. In the second task, the experimenter drew a dot in the sand in front of the seated participant and told them that this dot represented “today” (or alternative point in time). The participant was next asked to draw dots representing “tomorrow” and “yesterday” (or their equivalents). Participants were then rotated either 90° or 180° (whichever was least awkward in the experimental context) to arrange the remaining cards and dots. These experiments are also described in more detail by Boroditsky et al. (2008).

Boroditsky and Gaby (2010) ran the same pair of experiments with English speakers in California as well as speakers of four indigenous languages (including Kuuk Thaayorre) in Pormpuraaw. They found that the Pormpuraawan group tended to arrange the cards from east-to-west, unlike the English speaking participants who without exception represented time from left-to-right. These findings show the dominant frame of reference used in describing space (absolute in Kuuk Thaayorre, relative in English) to covary with the frame of reference employed in representing time (absolute in Kuuk Thaayorre, relative in English). But they do not speak to a causal link between the two. It may be that the habits of thought built through the frequent use of absolute spatial language lead consultants to apply the absolute frame to time in solving experimental tasks. But an equally plausible hypothesis is that Pormpuraawans live in a cultural and physical environment that encourages them to attend to geographical cues and to store them in terms of the cardinal directions. This attention to cardinal directions would then be the source of: (1) their complex linguistic encoding; (2) their prominence in discourse; and (3) their employment in improvised representations of time such as in the experimental tasks.

The present study aims to tease apart these potential causal factors by contrasting two small groups of ethnic Thaayorre living in Pormpuraaw, the first group (*n* = 6) being bilingual in Kuuk Thaayorre and English, the second group (*n* = 3) comprising monolingual speakers of English. The English-monolinguals are in other respects extremely similar to the Kuuk Thaayorre speaking cohort in terms of age, upbringing, level of education, and current employment. Indeed, each of the English-monolingual Pormpuraawans can be matched to a Kuuk Thaayorre speaking participant with the same employment status (e.g., one pair being retired, another working in garbage collection). Participants ranged in ages between 45 and 75, although exact age was hard to determine in two cases. Kuuk Thaayorre speaking participants were instructed in Kuuk Thaayorre by the author, but some follow-up questions asked in English were also responded to in English.

## Results

**Figure 1:**
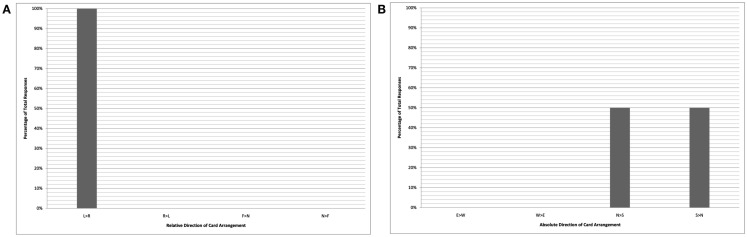
**English-monolinguals (card task)**. Chart shows relative coding of data **(A)** and absolute coding of data **(B)**. Key **(A)**: L > R “left-to-right”; R > L “right-to-left”; F > N “far-to-near”; N > F “near-to-far.” Key **(B)**: E > W “east-to-west”; W > E “west-to-east”; N > S “north-to-south”; S > N “south-to-north.”

Figure 1 plots data from the English-monolingual Pormpuraawans, who uniformly represent time as flowing from left-to-right, their performance indistinguishable from that of the Californian English speaking group of Boroditsky and Gaby (2010). These data are analyzed according to the participant-centric relative frame in the left-hand column A (with arrangements coded as left-to-right, right-to-left, far-to-near, and near-to-far), and analyzed according to the absolute frame in the right-hand column B (with arrangements coded as east-to-west, west-to-east, north-to-south, and south-to-north). Because these participants were tested while facing east (50% of the time) or west (50% of the time), their exclusively left-to-right arrangements show a 50/50 split between north-to-south and south-to-north directionality when analyzed from an absolute perspective.

**Figure 2:**
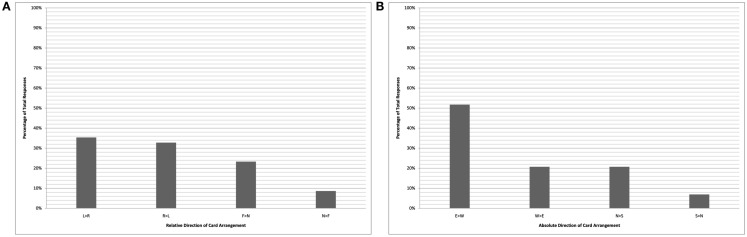
**Kuuk Thaayorre speakers (card task)**. Chart shows relative coding of data **(A)** and absolute coding of data **(B)**. Key **(A)**: L > R “left-to-right”; R > L “right-to-left”; F > N “far-to-near”; N > F “near-to-far.” Key **(B)**: E > W “east-to-west”; W > E “west-to-east”; N > S “north-to-south”; S > N “south-to-north.”

**Figure 3:**
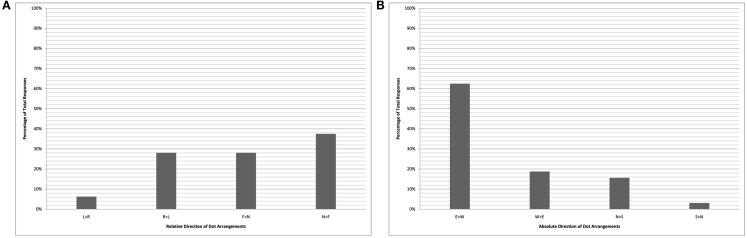
**Kuuk Thaayorre speakers (dot task)**. Chart shows relative coding of data **(A)** and absolute coding of data **(B)**. Key **(A)**: L > R “left-to-right”; R > L “right-to-left”; F > N “far-to-near”; N > F “near-to-far.” Key **(B)**: E > W “east-to-west”; W > E “west-to-east”; N > S “north-to-south”; S > N “south-to-north.”

**Figure 4:**
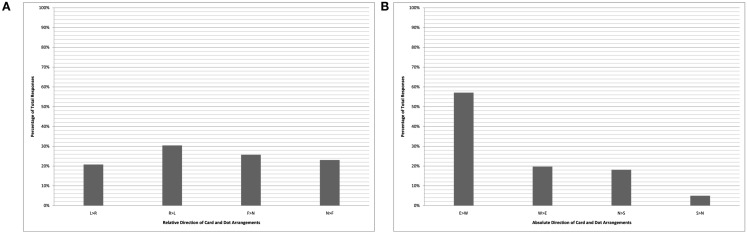
**Kuuk Thaayorre speakers (card + dot task)**. Chart shows relative coding of data **(A)** and absolute coding of data **(B)**. Key **(A)**: L > R “left-to-right”; R > L “right-to-left”; F > N “far-to-near”; N > F “near-to-far.” Key **(B)**: E > W “east-to-west”; W > E “west-to-east”; N > S “north-to-south”; S > N “south-to-north.”

The pair of charts in Figure 2 plot the performance of the bilingual cohort on the card-arrangement task, while the Figure 3 charts plot the bilinguals’ performance on the dot-drawing task (English-monolinguals were not tested on the dots task). A clear bias in favor of east-to-west representations is seen in the right-hand absolute analyses of both sets of data (labeled “B”). Due to an imbalance in the number of trials completed facing each of the four directions, there is an apparent (though illusory) bias against near-to-far card arrangements and against left-to-right dot drawings by the bilingual cohort. When these data are aggregated across the tasks (Figure 4), there is a roughly even distribution among the relative directions (left-to-right, right-to-left, near-to-far, and far-to-near), in stark contrast with the 100% left-to-right arrangements of the English-monolinguals.

The contrast between the representations of the Kuuk Thaayorre speaking Pormpuraawans, on the one hand, and of the English-monolingual Pormpuraawans, on the other, points to language as (co-) constitutive of their conceptualization of time, as will be explored further in the next section.

## Discussion

The two tasks show that Pormpuraawans who speak Kuuk Thaayorre tend to arrange time from east-to-west, while Pormpuraawans who speak only English arrange time exclusively from left-to-right. This correlates with a dominant absolute frame of reference in Kuuk Thaayorre and a dominant relative frame of reference in English. And yet neither of the Pormpuraawan groups can be claimed to think exactly as they speak in this regard. The Kuuk Thaayorre speakers do not speak of time as moving from east-to-west, although they may represent it that way. Nor do English-monolingual Pormpuraawans (or any other English speaking group) speak of time as moving from left-to-right, although they represent it that way. Three factors – the third of which may be particular to the Pormpuraawan context – complicate a causal relationship between temporal thought and language, as follows:

1. It is language use, not language knowledge, that constructs habits of thought;
2. Language is not the only influence on thought;
3. Pormpuraawan representations of time reflect representations of space and not the language of time.

The following sections address each of these factors in turn.

### Parole, not langue

It is widely accepted that language helps shape mental representations by encouraging its speakers to develop habits of thought (cf., Slobin, 1996). But it is worth emphasizing that it is the *use* a linguistic system is put to – and not the linguistic system *per se* – that feeds these habits. Habits are born out of repetition, it is not enough for a language to possess a term or set of terms if its speakers do not often use them. To wit, English possesses terms for the cardinal directions, but its speakers do not use them frequently or across a wide range of contexts (e.g., for small-scale arrangements). Egocentric terms in the relative frame of reference dominate English discourse, and accordingly English speakers have been shown to build egocentric mental models of space (e.g., Levinson, 2003; Majid et al., 2004). Non-linguistic representations should not be influenced by language, then, but by the linguistic culture of a community: how a language is put to use, including how often a particular term or structure is uttered as well as the full range of associations it receives in context (cf., Slobin, 1996).

The boundary between linguistic culture and culture more generally is, of course, fuzzy. Language is learned in a cultural context, culture is – in part – linguistically transmitted. When Thaayorre children learn to attend to their geographic surrounds, learn how locations relate to one another independently of an external viewer, and so on, they learn both by observing others’ behavior and by listening to their utterances. For this reason, it may be somewhat misleading to consider the English-monolinguals fully immersed in Thaayorre culture. But any cultural knowledge they lack must be linguistically transmitted, and can therefore be ultimately attributed to language, broadly defined.

Franz Boas famously championed the investigation of language as a window to culture. It’s not just anthropologists who learn about culture by investigating language; all members of a culture become so in part through their acquisition of that language.

### Language, its conspirators and competitors

Clearly, language is not alone in shaping non-linguistic representations, it must jostle for position against a range of conspirators and competitors. In the case of spatial representations of time, the powerful influence of writing direction (which may have been influenced by language historically, but is now learned entirely independently) has been amply demonstrated for a number of speech communities. For example, Hebrew speaker/writers have been shown to represent time as flowing from right-to-left (e.g., Fuhrman and Boroditsky, 2010), while Mandarin speaker/writers employ top-to-bottom representations (e.g., Boroditsky, 2001; Boroditsky et al., 2011) and English speaker/writers employ left-to-right representations (e.g., Tversky et al., 1991; Boroditsky, 2001). Crucially, a number of studies demonstrate participants’ representations of time to mirror writing direction even when this conflicts with the dominant metaphorical schema in language (e.g., Bergen and Chan Lau, 2012; de Sousa, 2012).

So what role does writing direction play in shaping representations of time amongst the Thaayorre? The English-monolingual and Kuuk Thaayorre speaking groups in this study do not differ overall in their respective levels of literacy. All participants are able to read and write, but do not use these skills frequently in day to day life. It has not been possible to acquire detailed data on the teaching of literacy during the period our participants attended school. The highest level of education (at Batchelor Institute of Indigenous Tertiary Education, formerly Batchelor College) was obtained by a member of the Kuuk Thaayorre speaking cohort and did not obviously affect his performance on the two tasks (e.g., by conditioning left-to-right arrangements). And yet, writing direction seems to have been formative of temporal representations for only one of the participant groups. The English-monolinguals in this study used the left-to-right axis exclusively, which is consistent with writing direction and not explained by English temporal metaphors (which primarily invoke the sagittal axis). The Kuuk Thaayorre speakers, in contrast, employed a range of different representations, most frequently invoking the east-to-west axis which is fundamentally incompatible with any viewer-oriented script. Why should writing direction play such an unequal role in the two cases? Let us consider two alternative explanations for this fact.

Firstly, we might suppose that participants must select a frame of reference to work within prior to developing a spatial representation of time. Habits of language use are likely to play a key role here. Speakers used to organizing the world in terms of absolute cardinal directions are more likely to choose an absolute frame for arranging cards or dot points. Speakers who habitually organize the world in terms of left and right are likely to favor a relative frame. Once a relative solution is adopted, literacy may determine (or at least strongly suggest) one directionality over another (in this case left-to-right rather than right-to-left, near-to-far, or far-to-near along the sagittal axis). But if an absolute frame is adopted, the literacy bias becomes irrelevant since it is inherently anchored to a relative viewer’s perspective. Instead, the arc of the sun as it is perceived to travel across the sky is an ideal model of the time/space nexus.

Alternatively, we might suppose that writing direction serves as a valid model for spatial representation for both participant groups, but that it must compete against others, with the winning candidate determined by frequency (cf., Bybee, 2010). English speakers may potentially employ each of the three frames of reference when speaking about space. They employ front-to-back and back-to-front metaphors for time in speech, and construct spatial representations of time from left-to-right, top-to-bottom, and in clockwise circles (e.g., timelines, cartoons, clocks, and other artifacts). But Pormpuraaw is far less saturated with such artifacts and imagery than most English speaking environments. Furthermore, the Thaayorre make little use of terms for the cardinal directions when speaking English, even when translating Kuuk Thaayorre texts replete with such terms (cf., Gaby, 2011). So we might suppose encounters with the written word to rank fairly highly amongst the competing representational modes for the English-monolingual group. But for the Kuuk Thaayorre speakers, these all pale in comparison with the frequency of absolute directional terms in Kuuk Thaayorre discourse.

Lastly, we are faced with the puzzle of why the English-monolinguals’ responses should be so much more uniform than those of the Kuuk Thaayorre/English bilinguals, who exhibited both intra-individual and inter-individual variability. Since the experimental tasks were explained to the English-monolinguals in the experimenters’ first language, it is possible that the instructions were clearer than for the Kuuk Thaayorre group (who received instruction in Kuuk Thaayorre or, in some cases, a mixture of Kuuk Thaayorre and English). We might alternatively – or additionally – account for the mixed strategies adopted by Kuuk Thaayorre speakers in terms of their bilingualism. This group must contend with competition between the two candidate frames of representation (the absolute frame favored by Kuuk Thaayorre and any other indigenous languages they are fluent in, the relative frame favored by English), as well as literacy and other representational practices (e.g., in the community’s store, post office and church).

### Representations of time are parasitic on representations of space

This study shows the link between language and non-linguistic representations of time to be indirect, mediated by representations of space. This points to there being at least two distinct components of the space -to- time mapping. Firstly, the frame of reference most often invoked in spatial reference creates habits of thought, habits that are either reinforced or diminished by other experiences of space and spatial representations. Secondly, there is a broad domain mapping from space to time. This mapping is both fed and reflected by the lexical polysemies noted under The Language of Time, but I would not expect it to be dependent on the presence of linguistic ambiguity and metaphors. The precise nature of non-linguistic representations of time is then shaped by the representations of space imported through the space to- time domain mapping. The frame of reference favored in spatial representations, both linguistic and non-linguistic, thus emerges in non-linguistic representations of time.

## Conclusion

The way people conceptualize time is shaped by a range of external influences, both linguistic and non-linguistic. This study has investigated the influence of language on certain spatial representations of time by testing two groups of Pormpuraawans who differ chiefly in their fluency in Kuuk Thaayorre, a language with a dominant absolute spatial reference system. The respective performances of the two groups support the idea that linguistic culture influences the construction of non-linguistic forms of representation. This in turn is suggestive of differences in habitual thought between speakers of different languages. Specifically, a linguistic culture that makes frequent use of terms for cardinal directions requires speakers of that language to attend to directional cues and to store them in memory. This absolute representation may then be projected onto other domains, such as time. A linguistic culture that privileges the relative frame leads speakers to interpret spatial configurations in terms of their own perspective, which may be likewise applied in construing time. This study thus finds spatial representations of time to be structured according to the frame of reference dominant in the language of the source domain (space), not the target domain (time).

## Conflict of Interest Statement

The author declares that the research was conducted in the absence of any commercial or financial relationships that could be construed as a potential conflict of interest.
